# A Remarkable Case

**Published:** 1879-01

**Authors:** 


A Remarkable Case.—Dr. Hoffmann, in a Vienna medical journal, relates the following remarkable case: A man, aged forty, fired a pistol shot at himself in the region of the left breast. A skin-burn resulted of the size of the palm of the hand, but no rupture of the continuity of the external skin. Beneath this there was an effusion of blood; the costal cartilage was broken. In the pericardium lay a pound and a half of blood; and at the apex of the heart, on each side of the longitudinal sulcus, was a rent of the muscular fibers, extending into the cavities of the ventricles.

